# Iron metabolism is disturbed and anti-copper treatment improves but does not normalize iron metabolism in Wilson’s disease

**DOI:** 10.1007/s10534-021-00289-x

**Published:** 2021-02-08

**Authors:** Grażyna Gromadzka, Diana Wierzbicka, Tomasz Litwin, Adam Przybyłkowski

**Affiliations:** 1 Faculty of Medicine, Collegium Medicum, Cardinal Stefan Wyszyński University in Warsaw, Wóycickiego Str. 1/3, 01-938 Warsaw, Poland; 2 Second Department of Neurology, Institute of Psychiatry and Neurology, Sobieskiego Str. 9, 02-957 Warsaw, Poland; 3 Department of Gastroenterology and Internal Medicine, Medical University of Warsaw, Banacha Str. 1a, 02-097 Warsaw, Poland

**Keywords:** Iron, Copper, Wilson disease

## Abstract

Wilson’s disease (WD) is a rare hereditary disorder of copper metabolism. Some data suggest that iron metabolism is disturbed in WD and this may affect the course of the disease. The current study aimed to determine whether anti-copper treatment could affect iron metabolism in WD. One hundred thirty-eight WD patients and 102 controls were examined. Serum ceruloplasmin and copper were measured by colorimetric enzyme assay or atomic adsorption spectroscopy, respectively. Routine and non-routine parameters of iron metabolism were measured by standard laboratory methods or enzyme immunoassay, respectively. WD patients, both newly diagnosed and treated, had less serum copper and ceruloplasmin than controls (90.0, 63.0, 22.0 mg/dL, respectively, p < 0.001); in the treated patients blood copper and ceruloplasmin were lower than in untreated patients (p < 0.001). Untreated patients (n = 39) had a higher median blood iron (126.0 vs 103.5 ug/dL, p < 0.05), ferritin (158.9 vs 47.5 ng/mL, p < 0.001), hepcidin (32, 6 vs 12.1 ng/mL, p < 0.001) and sTfR (0.8 vs. 0.7 ug/mL, p < 0.001) and lower blood transferrin (2.4 vs. 2.7 g/L, p < 0.001), TIBC (303.0 vs 338.0 ug/dL, p < 0.001), hemoglobin (13.1 vs 13.9 g/dL, p < 0.01) and RBC (4.3 vs. 4.6, p < 0.002) than controls. Treated patients (n = 99) had a significantly lower median iron (88.0 vs. 126.0 ug/dL, p < 0.001), ferritin (77.0 vs. 158.9 ng/mL, p < 0.005) and hepcidin (16.7 vs. 32.6 ng/mL, p < 001) and higher transferrin (2.8 vs. 2.4 g/L, p < 0.005), TIBC (336.0 vs 303.0 ug/dL, p < 0.001), RBC (4.8 vs. 4.3 M/L, p < 0.001) and hemoglobin (14.4 vs. 13.1 g/dL, p < 0.001) than untreated; the median iron (p < 0.005) was lower, and ferritin (p < 0.005), RBC (p < 0.005) and hepcidin (p < 0.002) were higher in them than in the control group. Changes in copper metabolism are accompanied by changes in iron metabolism in WD. Anti-copper treatment improves but does not normalize iron metabolism.

## Introduction

Wilson’s Disease (WD) (Online Mendelian Inheritance in Man [OMIM] Accession Number # 2779000, also known as hepato-lenticular degeneration, is a rare autosomal recessive disease that mainly manifests with hepatic or neuropsychiatric symptoms. More than 500 mutations have been identified in the *ATP7B* gene that cause intracellular copper transporter ATP-ase 7B (ATP7B) [OMIM * 606882] dysfunction leading to the accumulation of copper mainly in the liver and brain, but also in other organs such as the kidney and brain. In most cases the first symptoms of WD appear in childhood, but sometimes they can appear later even after the age of 60 (Mulligan and Bronstein 2020; Ala et al. 2007; Roberts and Schilsky 2008; Terada et al. 1998; Merle et al. 2007).

Copper and iron metabolism cross over different metabolic pathways. Therefore, clinical symptoms in patients with Wilson’s disease can be expected to be due to impaired copper as well as iron metabolism. This may be a consequence of the reduced production and activity of ceruloplasmin, an iron oxidase. Accumulation of copper and iron in the liver was observed in the experimental WD model (Chandarana et al. 2009; Koizumi et al. 1998; Huster et al. 2006; Park et al. 2006). Several human studies have also suggested that in WD iron metabolism may be impaired, and iron accumulation in the liver and/or brain tissue may worsen the clinical course of the disease (Litwin et al. 2013; Pfeiffenberger et al. 2011; Medici and Weiss 2017; Dusek et al. 2017).

Copper-iron interaction in WD should be clarified to better understand disease patomechanism and improve patient treatment. This study is the first that analyzes many parameters of iron metabolism in a relatively large population of patients with Wilson’s disease and compares the value of these parameters between untreated patients undergoing decoppering treatment as well as healthy controls.

## Patients and methods

A total of 138 patients with WD, including 39 previously untreated (newly diagnosed with WD) and 99 treated at the Institute of Psychiatry and Neurology in Warsaw, Poland. All patients met WD 8 diagnostic criteria. International Conference Wilson’s disease and Menkes’ disease (Ferenci et al. 2003). The median duration of treatment in the treated group was 48.5 months (IQR 47.0; range 6–168). Diagnosis of WD was based on the assessment of clinical symptoms (Gromadzka and Czlonkowska 2011; Gromadzka et al. 2011, 2015), abnormal copper metabolism (decreased serum ceruloplasmin (Cp) and serum copper, increased 24-h copper excretion in urine), presence of the Kayser–Fleischer ring and genetic test. In the case of an uncertain diagnosis, the inclusion of Cu64 in ceruloplasmin was measured after 24 and 48 h. All laboratory tests for copper metabolism were performed in the same laboratory using the same standardized methods. The DNA analysis for the three most frequent mutations of *ATP7B* detected in the Polish population was performed using polymerase chain reaction—restriction fragments length polymorphism method (PCR–RFLP) or—if this method did not confirm the presence of two pathogenic *ATP7B* mutations—the DNA sequencing was performed, as previously described (Gromadzka et al. 2005, 2011). Such diagnostic strategy allowed to obtain following results: 70 WD patients (50.7%) were homozygous for the c. 3207C > A [p.H1069Q] mutation, 51 (36.9%) had the c. 3207C > A [p.H1069Q] mutation in one allele and other rare mutation in the second one, and 17 subjects (12.3%) had rare mutations in both *ATP7B* alleles. According to the manner in which the disease was manifested, patients were grouped as hepatic or neuropsychiatric. Patients were classified as hepatic when they showed symptoms of chronic or acute liver disease (increased liver enzymes with increased bilirubin in the blood and abnormal INR and/or changes in liver echogenicity; signs of portal hypertension, decompensated liver cirrhosis or acute liver failure) without neuropsychiatric symptoms. Patients were classified as neuropsychiatric when liver disease was accompanied by neurological symptoms such as tremor, dystonia, ataxia, dysarthria, stiffness or mental symptoms, including behavioral disorders, depression, manic psychosis, or cognitive impairment. Several patients were diagnosed as presymptomatic (they were identified thanks to family studies of index cases).

The control group consisted of 102 healthy volunteers with an average age of 35.6 ± 10.5 years who had no family history of WD, diagnosed liver disease, neurological or psychiatric disease, chronic inflammatory disease or infectious disease. Control people were recruited from hospital staff and their families. The three most common *ATP7B* mutations in the Polish population were absent in these individuals, as documented by DNA sequence analysis using the methods previously described (Gromadzka et al. 2011). Detailed criteria for inclusion and exclusion in the study group are presented in Table 1

**Table 1:** Inclusion and exclusion criteria for the study

	Patients with WD	Control group
Inclusion criteria	Established WD diagnosis Age over 18 years Written consent to participate in the study	Absence of any disease Age over 18 years Written consent to participate in the study
Exclusion criteria	Prevalence of systemic diseases: cancer, current infection, hepatitis virus infection History of blood transfusion performed up to a month before qualifying for the study History of iron supplementation up to a year before qualifying for the study Alcohol abuse Lack of consent to participate in the study	Prevalence of systemic diseases: cancer, current infection, hepatitis virus infection History of blood transfusion performed up to a month before study History of iron supplementation up to a year before qualifying for the study Alcohol abuse Lack of consent to participate in the study Occurrence of phenotypic traits of WD The presence of the *ATP7B* mutation

Blood samples were taken prospectively from all subjects for laboratory analysis. Biological material was collected as separate portions for use in individual measurements to avoid repeated freezing and thawing cycles. Samples were stored at − 70 °C. Samples were used for testing immediately after thawing. All study participants gave written informed consent to participate in this study. The study protocol was approved by the local Ethics Committee and was conducted in accordance with the Code of Ethics of the World Medical Association (Helsinki Declaration) for human experiments.

Serum ceruloplasmin was measured by an enzymatic colorimetric assay as described earlier (Ravin 1961). Serum copper was determined by atomic adsorption spectroscopy using a Shimadzu AA660 instrument.

Routine iron metabolism parameters such as ferritin, transferrin, total iron binding capacity (TIBC), red blood cells (RBC) and hemoglobin (HGB) were measured by standard laboratory methods.

Hepcidin, lactoferrin and soluble transferrin receptor were measured using ELISA kits. Serum lactoferrin was evaluated using the Human Lactoferrin ELISA Test Kit (catalogue number EL20111-1; Test Pro) with a test range of 0.625–40,000.0 ng/mL. Serum hepcidin level (Hepc) was tested using a hepcidin-associated immunosorbent assay kit (cat. No. SEB979Hu; Cloud Clone Corporation) with a test range of 62.5–4000.0 pg/mL. Serum soluble transferrin receptor (sTfR) was tested using a sandwich enzyme-assay (cat. No. RD194011100; BioVendor;) with a test range of 0.4–2.0 µg/mL.

### Statistical analysis

The normality of the analysed continuous variables was determined by Kolmogorov–Smirnov and Lilliefors tests. Data are presented as the mean and standard deviation if they were normally distributed, or as the median and interquartile range if the variables were not normally distributed. Student’s *t* test was used to compare two groups of normally distributed data. The Mann–Whitney U test was used to compare variables that were not normally distributed. Qualitative data is defined as percentage ratios. Comparisons between groups were performed using a Chi square test with Yates correction, if applicable, or Fisher’s exact test, when the Chi square test hypothesis was not met. Significance was set at p < 0.05. All statistical analyses were performed using the STATISTICA 10.0 software (StatSoft PL, Krakow, Poland).

## Results

Patient baseline characteristics, including age, type of WD manifestation, presence of the Kayser-Fleisher ring (KF) and copper metabolism parameters are shown in Table 1. The average age of WD patients was 36.7 × 11.3 years, which was similar to the age of control (33.5 vs. 5.9 years, p < 0.09). The two groups were similar in terms of gender distribution (chi2 0.003, df 1, p < 0.95). Patients included in the untreated and treated groups did not differ in age and sex (Table 2).

**Table 2:** Clinical and laboratory characteristics of studied population

Characteristics	Controls	Untreated patients	Patients on treatment	p
Number of persons (n)	102	39	99	–
Age at onset of WD (median (IQR))	31.0 (8.0)	34.5 (18.5)	33.0 (14.0)	(a) < 0.07 (b) < 0.18 (c) < 0.84
Sex. female/male (n (%))	51/51 (50/0)	23/16 (59/41)	41/58 (41/59)	(a) < 0.34 (b) < 0.22 (c) < 0.06
Patients treated with D-penicylamine/zinc sulphate (n (%))	–	–	50/49 (50.5/49.5)	–
Time of treatment, months (median (IQR))	–	–	54.5 (58.0)	–
Clinical phenotype of WD (n (%))	–			
Hepatic		11 (34.4)	25 (25.2)	< 0.58
Neuropsychiatric		18 (56.2)	65 (65.7)	
Presymptomatic		3 (9.4)	9 (9.1)	
No data		7	–	

*IQR* interquartile range, *SD* standard deviation

*p values for the comparison between untreated and treated patients with WD

WD patients, both untreated and receiving therapy, had significantly lower serum copper (63.0 vs 22.0 vs 90.0 µg/dL, p < 0.001) and ceruloplasmin than controls (15.3 vs 5.4 vs 31.8 mg/dL, p < 0.001, Table 3). The blood concentration of copper and ceruloplasmin in treated patients were lower than in untreated patients, as shown in Table 3.

Untreated patients had statistically significant higher concentration of ferritin than patients on treatment (158.9 vs 77.0 ng/mL, p < 0.005). Patients form both groups however had higher ferritin concentration than controls 47,5 ng/mL, p < 0.005, Table 4).

**Table 3:** Markers of copper metabolism in WD patients and healthy controls

Parameter	Controls (n = 102)	Untreated patients (n = 39)	Patients on treatment (n = 99)	p
Serum copper (µg/dL) (median (IQR))^**f**^	90.0 (26.0)	63.0 (17.0)	22.0 (35.0)	(a) < 0.001 (b) < 0.001 (c) < 0.001
Serum ceruloplasmin (mg/dL) (median (IQR))	31.8 (10.7)	15.3 (8.0)	5.4 (6.7)	(a) < 0.001 (b) < 0.001 (c) < 0.001

*IQR* interquartile range

^**f**^Reference values: 25.0–45.0 mg/dL for serum ceruloplasmin; 70.0–140.0 µg/dL for serum copper

(a) p for the comparison between untreated and treated patients; (b) p for the comparison between treated patients and controls; (c) p for the comparison between untreated patients and controls

Iron concentration was noteworthy lower in treated patients than in treatment naive (88.0 vs 126.0 ug/dL, p < 0.001, Table 4). Treated patients presented lower iron values than controls (88.0 vs 103.5 µg/dL p < 0.002). Hepcidin concentration was highest in untreated patients, lower in treated group (32.6 vs 16.7 ng/mL, p < 0.001) and lowest in controls (12.1 ng/mL, p < 0.001). Red blood cells count and haemoglobin concentration were highest in patients on treatment, lower in controls (RBC 4.6 vs 4.8 M/L p < 0.005, hemoglobin 13.9 vs 14,4 g/dL p < 0.064) and lowest in patients without treatment (RBC 4.3 M/L, hemoglobin 13.1 g/dL). Lactoferrin concentration did not differ significantly between study groups.

Transferrin concentration was higher in treated patients than untreated (2.8 vs 2.4 g/L, p < 0.005), it was also higher in controls than in untreated group (2.75 vs 2.4 g/L p < 0.001), meanwhile soluble transferrin receptor concentration was lower in controls than in the group of untreated patients (0.7 vs 0.8, p < 0.001). The two groups of patients did not differ in term of sTfR concentration (Table 4). Total iron binding capacity was highest in controls, significantly lower in untreated WD population (338.0 vs 303.0 p < 0.001), with notably difference between treated group and untreated group (336.0 vs 303.0, p < 0.001, Table 4).

**Table 4:** Markers of iron metabolism in WD patients and healthy controls

Parameter	Controls (n = 102)	Untreated patients (n = 39)	Patients on treatment (n = 99)	p
Ferritin (ng/mL) (median (IQR))	47.5 (83.0)	158.9 (257.9)	77.0 (108.0)	(a) < 0.005 (b) < 0.005 (c) < 0.001
Hemoglobin (g/dL) (median (IQR))	13.9 (2.0)	13.1 (1.4)	14.4 (2.1)	(a) < 0.001 (b) < 0.064 (c) < 0.010
Hepcidin (ng/mL) (median (IQR))	12.1 (15.8)	32.6 (47.2)	16.7 (26.0)	(a) < 0.001 (b) < 0.002 (c) < 0.001
Iron (µg/dL) (median (IQR))	103.5 (52.0)	126.0 (58.0)	88.0 (51.0)	(a) < 0.001 (b) < 0.005 (c) < 0.052
Lactoferrin (ng/mL) (median (IQR))	295.5 (110.0)	326.0 (221.0)	289.0 (166.0)	(a) < 0.091 (b) < 0.738 (c) < 0.084
Red blood cells (M/L) (median (IQR))	4.6 (0.7)	4.3 (0.9)	4.8 (0.7)	(a) < 0.001 (b) < 0.005 (c) < 0.002
TIBC (µg/dL) (median (IQR))	338.0 (54.0)	303.0 (103.8)	336.0 (80.0)	(a) < 0.001 (b) < 0.075 (c) < 0.001
sTfR (µg/mL) (median (IQR))	0.7 (0.3)	0.8 (0.5)	0.8 (0.5)	(a) < 0.159 (b) < 0.106 ((c) < 0.001
Transferrin (g/L) (median (IQR))	2.75 (0.54)	2.4 (0.8)	2.8 (0.7)	(a) < 0.005 (b) < 0.722 ((c) < 0.001

*IQR* interquartile range

(a) p for the comparison between untreated and treated patients; (b) p for the comparison between treated patients and controls; (c) p for the comparison between untreated patients and controls; IQR, interquartile range

## Discussion

The presented results showed that iron metabolism is disturbed in WD. We document that both newly diagnosed and treated patients present iron metabolism abnormalities that were more apparent in the untreated group. Before start of treatment, patients with WD had higher concentration of iron, ferritin and hepcidin, and lower blood transferrin, TIBC, HGB, and RBC than controls. In our opinion, most of these abnormalities may results from hepatitis (Morgan and Philip 2002). Inflammation is associated with changes in the synthesis of so-called acute phase proteins (APP), whose production increases (positive APP) or decreases (negative APP) during inflammation. Ferritin and hepcidin belong to the positive APP group, the production of which increases, while transferrin represents negative APP, the concentration of which decreases in inflammation. Since circulating iron is bound mainly to transferrin, the TIBC value is an indirect measure of the concentration of this protein .

In our view, lower than in the control group, red blood cell counts and hemoglobin levels in untreated patients may be due to copper toxicity. An excess of free copper in the blood can stimulate the production of reactive oxygen species, oxidation of red blood cells, and finally impaired function and shortened survival (Ward et al. 2014; Morris et al. 1995; Harris et al. 1998).

There was no difference in blood lactoferrin between untreated patients and the control group. This was an unexpected result because the concentration of this protein increases during excessive iron intake as well as inflammation. However, as Brock suggests, excess lactoferrin is removed very quickly from the blood (24–48 h) by macrophages and monocytes, and then the iron associated with lactoferrin is converted to ferritin (Adlerova et al. 2008; Brock 2002).

We observed that transferrin, hemoglobin, TIBC, lactoferrin and sTfR normalized on WD treatment. The hepcidin and ferritin remained elevated, but both were lower in the treatment group than in the untreated group. The reduction of ferritin and hepcidin and the increase in TIBC in treated patients may be interpreted as a marker of resolution of hepatitis due to decoppering treatment. Lower hepcidin concentration in treated patients than in untreated may also be associated with normalization of iron metabolism resulting from decoppering treatment and therefore—a mechanism to prevent iron overload including hepcidin activity, may no longer needed. Unexpectedly treated patients had higher red blood cell counts and lower iron levels than control and untreated patients. This may be due to greater erythropoietic activity or other—yet undiscovered—mechanisms.

We have not presented results of copper and iron status according to phenotype. In both clinical phenotype of the disease i.e. hepatic and neuropsychiatric liver is affected. We have performed such analysis- there was no significant differences between groups. The differences of copper and iron metabolism between female and male patients with WD have been analyzed in a separate study (Gromadzka et al. 2020).

We are aware that although we analyzed a relatively large number of patients, the number of untreated patients was small and probably due to this a few of the observed differences did not reach statistical significance. Therefore, more studies are needed to verify the presented results

In summary, our findings indicate that changes in copper metabolism are accompanied by changes in iron metabolism in WD; anti-copper drug treatment restores, but does not normalize iron metabolism in patients with WD. Further research is needed to determine the optimal methods for control and normalization of iron metabolism in patients with WD.

## Data Availability

All study data are available at request- contact to corresponding author.
